# Middlesex Hospital

**Published:** 1831-04

**Authors:** 


### HOSPITAL REPORTS.

### MIDDLESEX HOSPITAL.

### *Ulcerative Disease of the Rectum removed by Excision.*

Anne Kay, æt. thirty-eight, about two years since, began to experience considerable pain in the rectum, when the bowels acted. In a short time this pain increased in severity, and was attended with unpleasant sensations, extending round the loins and to the pubes, with a sense of weight on the sacrum, and some discharge from the bowels, coloured with blood. These symptoms, notwithstanding some occasional partial amendments, progressively increased in severity. In December, when she became a patient in the Middlesex Hospital, she suffered constant aching and shooting pain, aggravated on the passage of feces and when she attempted to stand up and walk, and attended with purulent and bloody discharge.

She is a married woman, and has had three children; the last, several years ago. Until the last six months, she continued regular, when, at the recurrence of the period, a profuse flow of blood took place; since then, there has been no uterine secretion.

Upon an examination of the rectum, it was felt to be indurated and ulcerated for the extent of two inches; but the finger could be passed beyond the hardened part, into perfectly healthy structure. Various remedies were tried for the relief of the disease, but ineffectually; the patient's sufferings continued to increase, and there appeared no probability that any remedies would arrest this progress: accordingly, Mr. Mayo determined to remove the diseased part.

The operation was performed on the 25th of February, in the following manner: The patient was laid upon her side, with the hips and knees bent. The fingers being then introduced into the rectum, the knife was plunged into the perineum, on one side of the bowel, and, an incision of some depth being thus made laterally, the dissection was continued forwards from thence, so as to separate the vagina from the rectum. The dissection was then continued entirely round the rectum, including half an inch of integument, with the sphincter muscle. By this means, a length of two inches and a half of the extremity of the rectum was separated from the adjacent parts: it was then cut off with scissors from the sound rectum above. The operation was performed slowly, and the vessels, about nine in number, were tied as they were divided. The patient lost about twelve or fourteen ounces of blood.

It is unnecessary to detail the subsequent treatment. The case has gone on most favorably. The patient observed, two hours after the operation, when the smart of the wound had begun to subside, that she felt entirely relieved of the pain and distress from which she had suffered so many months.

The appearance of the wound is singular: the extremity of the bowel is not more than half an inch from the cut edge of the skin, and the intervening granulations are healthy, and cicatrizing rapidly. The bowels act regularly once a day: the patient is aware, as before the disease began, of the presence of feces in the rectum; about five minutes after this sensation occurs, the bowels act, much in the same manner, she says, as formerly, although it is very clear that, at present, there can be nothing equivalent to a sphincter muscle at the anus: it is, however, far from impossible that, when the wound has cicatrized and contracted, she will have the power of retaining solid feces.

When the part removed was examined, it was found to include a third of an inch of healthy intestine; the disease extended from the anus full two inches along the bowel, which was thickened and indurated, and the middle inch of the diseased portion was in a state of ulceration.

In our last number (p. 275,) we gave a brief account of M. LISFRANC's mode of operating in cases of cancer of the rectum. We believe, the above is the first instance in which the operation has been performed in this country.

---

### *Amputation of both Legs after Mortification.*

JAMES HUNT, ætat. thirty-three, was admitted into the Middlesex Hospital, under the care of Mr. MAYO, on Saturday, the 22d of January. Both feet were in a state of gangrene, which extended to four or five inches above the ankle-joints: the gangrene had evidently ceased to spread for several days; for an ulcerated line separated the dead skin from the living. The integuments of the foot were, however, but partially discoloured, and vesication had taken place at a few points only. No blood flowed upon puncturing the feet; yet the toes still moved voluntarily, the living muscles pulling upon the dead tendons. The patient stated, that the feet had mortified from exposure to cold, while he was recovering from a fever.

At the time of his admission, he was much emaciated: his pulse was 120; he scarcely slept a moment; his back was sore from lying; but his tongue was clean, his appetite good, and he complained of little pain.

He was allowed meat and porter: the last appeared to heat him, and was discontinued the next day. His strength appeared to improve during the 23d, 24th, 25th, the three days following his admission. On the 26th, however, he had lost ground: he had passed a night of considerable pain; his countenance was anxious, his tongue slightly furred, and his appetite failed him. Nevertheless, the day after, he was again better: he had suffered less pain during the night, and his appetite had returned.

The feet were now quite black, and the cuticle had separated to a considerable extent. Under these circumstances, Mr. MAYO determined to avail himself of the present favorable condition of his patient, thinking that he had recovered sufficient strength to bear the removal of his limbs, and apprehensive of a change for the worse, which another twenty-four hours might bring about. Accordingly, Mr. Mayo amputated both limbs below the knee, on the afternoon of the 27th. Forty minims of laudanum were then administered to the patient, and he was put to bed. He had some sleep during that night; but the two following, he was delirious; the pulse 140.

He had hitherto taken broth repeatedly since the operation, but nothing more nutritious. On the fourth night, he became extremely low, the pulse intensely rapid, and he appeared to be sinking: some wine and water was cautiously given him, and repeated. The following day, his whole appearance was greatly improved.

His subsequent progress has been slow, but uniform, with the exception of two slight attacks of erysipelas, one occurring upon each stump, at an interval of three days apart. The stumps are now far advanced towards healing; his appetite has returned; he sleeps well, and improves in strength daily.

